# The landscape of cell regulatory and communication networks in the human dental follicle

**DOI:** 10.3389/fbioe.2025.1535245

**Published:** 2025-02-05

**Authors:** Jia-Ning Liu, Jiong-Yi Tian, Lu Liu, Yuan Cao, Xiao Lei, Xiao-Hui Zhang, Zi-Qi Zhang, Jun-Xi He, Chen-Xi Zheng, Chao Ma, Sheng-Feng Bai, Bing-Dong Sui, Fang Jin, Ji Chen

**Affiliations:** ^1^ State Key Laboratory of Oral & Maxillofacial Reconstruction and Regeneration, National Clinical Research Center for Oral Diseases, Shaanxi International Joint Research Center for Oral Diseases, Center for Tissue Engineering, School of Stomatology, The Fourth Military Medical University, Xi’an, Shaanxi, China; ^2^ State Key Laboratory of Oral & Maxillofacial Reconstruction and Regeneration, National Clinical Research Center for Oral Diseases, Shaanxi Clinical Research Center for Oral Diseases, Department of Orthodontics, School of Stomatology, The Fourth Military Medical University, Xi’an, Shaanxi, China; ^3^ Department of Oral Implantology, School of Stomatology, The Fourth Military Medical University, Xi’an, Shaanxi, China

**Keywords:** dental follicle, single-cell RNA sequencing, gene regulatory networks, cell–cell communication, vasculature, immune

## Abstract

**Introduction:**

The dental follicle localizes the surrounding enamel organ and dental papilla of the developing tooth germ during the embryonic stage. It can differentiate and develop to form the periodontal ligament, cementum, and alveolar bone tissues. Postnatally, the dental follicle gradually degenerates, but some parts of the dental follicle remain around the impacted tooth. However, the specific cellular components and the intricate regulatory mechanisms governing the postnatal development and biological function of the dental follicle have not been completely understood.

**Methods:**

We analyzed dental follicles with single-cell RNA sequencing (scRNA-seq) to reveal their cellular constitution molecular signatures by cell cycle analysis, scenic analysis, gene enrichment analysis, and cell communication analysis.

**Results:**

Ten cell clusters were identified with differential characteristics, among which immune and vessel-related cells, as well as a stem cell population, were revealed as the main cell types. Gene regulatory networks (GRNs) were established and defined four regulon modules underlying dental tissue development and microenvironmental regulation, including vascular and immune responses. Cell–cell communication analysis unraveled crosstalk between vascular and immune cell components in orchestrating dental follicle biological activities, potentially based on COLLAGAN-CD44 ligand–receptor pairs, as well as ANGPTL1-ITGA/ITGB ligand–receptor pairs.

**Conclusion:**

We establish a landscape of cell regulatory and communication networks in the human dental follicle, providing mechanistic insights into the cellular regulation and interactions in the complex dental follicle tissue microenvironment.

## 1 Introduction

The dental follicle is a layer of loose connective tissue that surrounds the developing tooth germ or the impacted tooth (Zeng et al., 2022). During the embryonic stage, the dental follicle surrounding the enamel organ and dental papilla is composed of ectomesenchymal cells that are derived from the neural crest cells (Zeng et al., 2022; Zhou et al., 2019). These cells, known as dental follicle cells (DFCs), are multipotent stem cells capable of differentiating into various cell types and finally to form the periodontal ligament, cementum, and alveolar bone tissues (J et al., 2021). In addition, the dental follicle is essential for tooth eruption as it regulates the resorption of bone to form an eruption pathway and provides the driving force for the tooth to move toward its functional position in the oral cavity (Shiyan et al., 2016; Wise, 2009). Postnatally, the dental follicle gradually degenerates, and the periodontal tissues differentiate and mature. Some parts of the dental follicle remain due to the impacted state (Yang et al., 2019). However, the specific cellular components and the intricate regulatory mechanisms governing the post-natal development and function of the dental follicle are not yet completely understood.

Single-cell RNA sequencing (scRNA-seq) has emerged as a powerful tool for dissecting cellular heterogeneity within complex tissues, allowing for a comprehensive understanding of cellular subpopulations and their functional roles (Vallejo et al., 2021; Wen et al., 2022). So far, scRNA-seq has been employed to investigate the human tooth germ derived from the developing third molar (Shi et al., 2021). A large number of immune cells were discovered in the tooth germ of the human third molar, highlighting the significant immune characteristics of the human third molar in the adult stage, and these cells regulate other dental cells through signaling pathways such as TGF-β, TNF, and IL-1 (Shi et al., 2021). Comparative analysis between human and mouse teeth revealed both parallels and divergences in tissue heterogeneity, emphasizing the molecular differences and species-specific cell subtypes (Krivanek et al., 2020). These findings underscored the utility of scRNA-seq in elucidating the intricate signaling networks that govern essential biological processes in human teeth postnatally.

The dental follicle, a reservoir of odontogenic mesenchymal stem cells, plays a pivotal role in tooth development and periodontal tissue regeneration (Yang et al., 2019; Guo et al., 2009). Under physiological conditions, these dental MSCs contribute to angiogenesis, a critical process in tissue regeneration (Li et al., 2022). Conversely, in pathological states, they participate in immune modulation, highlighting their dual role in maintaining homeostasis and tissue regeneration (Li et al., 2023). Moreover, vascular and immune cell components also collaborate and contribute to dental tissue homeostasis, but the specific mechanisms remain not fully understood (Zarubova et al., 2022). Understanding the transcriptomic landscape of the dental follicle is not only essential for uncovering the molecular regulatory mechanisms but also harnessing its regenerative potential (Zarubova et al., 2022; Azari et al., 2023). The ability to modulate the behavior of dental follicle cells offers a promising avenue for developing novel therapeutic strategies in regenerative dentistry and medicine. By identifying key regulatory nodes within the dental follicle transcriptome, we can potentially direct cells toward desired lineage commitments, enhancing vascularization, and immune modulation for tissue repair.

In this study, we aimed to explore the transcriptome heterogeneity, cell regulatory network, and cell–cell communication of the dental follicle to uncover the molecular signatures associated with its angiogenesis and immunomodulatory functions. We performed scRNA-seq analysis of the human dental follicle and revealed the cellular composition and heterogeneity of this tissue. We further identified specific regulons governing the progenitor cell destiny and discovered immune and vascular cell components with reciprocal signaling to maintain the dental follicle niche. Collectively, our results unravel the previous unrecognized cellular landscape of the dental follicle at the postnatal stage and provide a comprehensive understanding of the potential mechanisms governing cellular biological activities and cell communication to function synergistically, highlighting the potential intervention targets like COLLAGAN-CD44 and ANGPTL1-ITGA/ITGB ligand–receptor pairs to promote tissue regeneration.

## 2 Materials and methods

### 2.1 Data availability

The raw sequence data analyzed in this research have been uploaded into Genome Sequence Archive (Chen et al., 2021) in National Genomics Data Center (Xue et al., 2022), China National Center for Bioinformation/Beijing Institute of Genomics, Chinese Academy of Sciences, and the data under accession HRA008022 will be available on 2026-07-12 automatically or will be made available at https://ngdc.cncb.ac.cn/search/specific?db=hra&q=HRA008022 upon publication.

### 2.2 Human tissue harvest and preparation

**FIGURE 1:**
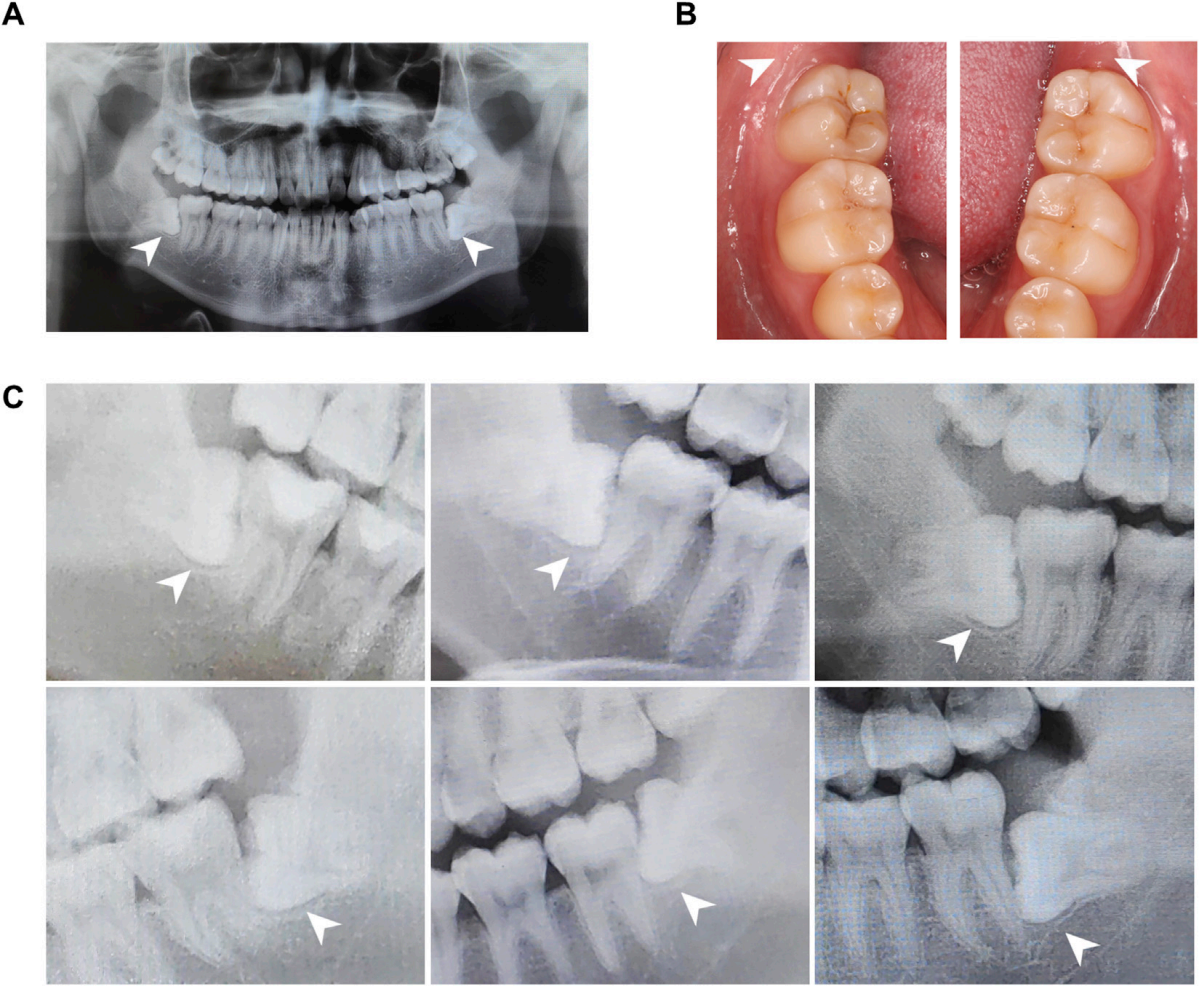
Panoramic radiographs and clinical views of the human dental follicle tissue. **(A)** A representative panoramic radiograph. **(B)**. Intraoral images illustrate non-inflammatory infiltration. **(C)**. Parts of panoramic radiographs of the human dental follicle tissue from a bite-wing perspective. Dental follicles from six wisdom teeth were identified without an abnormal low-density shadow.

All donors were patients in the School of Stomatology, The Fourth Military Medical University, and have signed informed consent to this study. Experimental procedures of human samples were approved by the Ethics Committee of The Fourth Military Medical University with the approval number IRB-REV-2022187. We collected dental follicle tissues from six impacted third molars of patients aged 18–22 years. Dental follicles were harvested based on inclusion criteria as follows: patients had no history of pain or infection around the impacted teeth, and clinical examination showed no swelling, redness, or tenderness in the pericoronal tissues of the third molars (Table 1; Figures 1A, B). All the patients were examined by oral panorama before surgery, and radiographic examination revealed no abnormal low- or high-density shadows around the crown (Figure 1C). By flap reflection, bone removal, tooth sectioning, and luxation, we obtained the third molars with attached dental follicle tissue, which were immediately immersed in 10% alpha-minimum essential medium (α-MEM; 12571-048, Invitrogen, United States) and delivered to the laboratory in an ice box.

**TABLE 1:** Clinical characteristics of participants.

Sample	Gender	Age	Tooth location	Pain or infection history	Redness or swelling	Low-density shadow on oral panorama
1	Female	20	48	No	No	No
2	Male	19	48	No	No	No
3	Male	20	48	No	No	No
4	Female	21	38	No	No	No
5	Female	20	38	No	No	No
6	Male	20	38	No	No	No

### 2.3 ScRNA-seq analysis

Dental follicles were obtained by cutting off soft tissues around the teeth. The tissues were sectioned into 2-mm^3^ pieces and rinsed twice with phosphate-buffered saline (PBS; P5493, Sigma-Aldrich, United States) before dental follicle tissues were initially dissociated into single cells using 0.02% Type I collagenase (17018029, Gibco, United States). Cells were barcoded with 10× gel beads and encapsulated in oil to form single-cell gel beads-in-emulsion (GEMs). Reverse transcription reactions were engaged barcoded full-length cDNA, followed by the disruption of emulsions using the recovery agent. Single-cell 3′ Reagent v3 Kits (1000268, 10× Genomics, United States) were used for scRNA-seq library construction. The sequencing was performed on Illumina Nova 6000 PE150 platform (Illumina, United States). Cell Ranger (version 7.0.1) software (10× Genomics, United States) was employed for quality control and through comparisons between reads to the genome by the spliced transcript alignment to a reference (STAR) aligner. The cells that did not meet the criteria were discarded: 1) gene numbers >200, unique multiplex index (UMI) > 1000 and log10GenesPerUMI >0.7; 2) the UMI of mitochondrial genes <15% and hemoglobin genes <5%. Subsequently, the DoubletFinder package (version 2.0.3) was used to identify potential doublets (McGinnis et al., 2019). To obtain the normalized gene expression data, library size normalization was processed using the NormalizeData function. Specifically, the global-scaling normalization method “LogNormalize” normalized the gene expression measurements for each cell by the total expression, multiplied by a scaling factor (10,000 by default), and log-transformed the results.

### 2.4 Dimensionality reduction and clustering

Top 2000 highly variable genes (HVGs) were calculated using the Seurat function FindVariableGenes (mean.function = FastExpMean, dispersion.function = FastLogVMR). Principal component analysis (PCA) was performed to reduce the dimensionality with RunPCA function. Graph-based clustering was performed to cluster cells according to their gene expression profile with the FindClusters function. Cells were visualized using a 2-dimensional t-distributed stochastic neighbor embedding (t-SNE) algorithm with the RunTSNE function. The FindAllMarkers function (test.use = presto) was used to identify marker genes of each cluster. Differentially expressed genes (DEGs) were selected using the function FindMarkers (test.use = presto). *P* value <0.05 and |log2foldchange| > 0.58 were set as the thresholds for significantly differential expression.

### 2.5 Cell type annotation

By employing the SingleR package (version 1.4.1) based on a public reference dataset, we calculate the correlation between the expression profiles of the cells to be identified and the reference dataset, assigning the most correlated cell type from the reference dataset to the cells in question, thereby largely eliminating subjective human factors. The principle of identification involves calculating the Spearman correlation between the expression profiles of each cell in the sample and each annotated cell in the reference dataset, selecting the cell type with the highest expression correlation in the dataset as the final identified cell type. Finally, we manually correct the cell types based on the characteristic expression genes of common cells according to the previous acknowledgments made by the authorities.

### 2.6 Cell proportion analysis

The cell abundance is determined based on the number of cells included in each cell population. Data visualization libraries plotAbundance in the R package (4.0.3) is used to create a stacked bar chart that represents the proportion of each cell population.

### 2.7 Cell cycle analysis

The cell cycle phase of individual cells was inferred by Seurat (Butler et al., 2018) with CellCycleScoring function. The function in the Seurat package is designed to calculate cell cycle scores to individual cells based on the expression of classic genes that are indicative of different phases of the cell cycle, particularly the S phase and the G2/M phase. Concisely, these marker gene sets are inversely correlated with their expression levels; cells that do not express these marker genes are likely to be in the G1 phase.

### 2.8 Single-cell regulatory network inference and clustering (SCENIC) analysis

The SCENIC analysis, a tool for reconstructing gene regulatory networks (GRNs) and identifying cell states, was performed by the motifs database for RcisTarget and GRNboost (SCENIC (Aibar et al., 2017) version 1.1.2.2, which corresponds to RcisTarget 1.2.1 and AUCell 1.4.1) with the default parameters. In detail, we identified potential targets for each transcription factor based on the co-expression relationships between genes. Next, we identified TF binding motifs over-represented on a gene list with the RcisTarget package. Lastly, the activity of each group of regulons in each cell was scored by the AUCell package.

To evaluate the cell-type specificity of each predicted regulon, we calculated the regulon specificity score (RSS) which was based on the Jensen–Shannon divergence (JSD), a measure of the similarity between two probability distributions. Specifically, we calculated the JSD between each vector of binary regulon activity which overlaps with the assignment of cells to a specific cell type (Suo et al., 2018). The connection specificity index (CSI) for all regulons was calculated with the scFunctions (https://github.com/FloWuenne/scFunctions/) package.

### 2.9 Gene enrichment analysis

Gene enrichment analysis, specifically Gene Ontology (GO) enrichment analysis, is widely used to test the enrichment of certain functions or characteristics within a set of genes. It compares genes with the GO database to identify significantly enriched GO terms, thereby inferring the functions and relationships of genes with other genes. GO terms are categorized into three main aspects, namely, molecular function, biological process, and cellular component, which provide a broad visualization of gene activities and help us extract biological meaning after numerous hypothesis tests accurately. Product properties of regulons from each CSI module were analyzed based on the GO dataset. GO enrichment analysis was performed using the oeCloud tools at https://cloud.oebiotech.com.

### 2.10 Cell communication analysis

CellChat (Jin et al., 2021) (version 1.1.3) R package was used for prediction of cell–cell interactions in the dental follicle. After creating a CellChat object using the createCellChat function, the data were preprocessed with the identifyOverExpressedGenes, identifyOverExpressedInteractions, and projectData functions. Potential ligand–receptor interactions were inferred according to the calculated results of the computeCommunProb, filterCommunication, and computeCommunProbPathway functions. Finally, the aggregateNet function was employed to aggregate the intercellular interaction network.

### 2.11 Immunofluorescence staining

For acquired human dental follicles, tissues were first fixed with 4% PFA for 12 h and dehydrated in 30% sucrose solution (Sigma-Aldrich, United States). Embedded in the O.C.T compound (4583, Sakura Finetek, United States), tissues were made to obtain 10-μm frozen sections by a freezing microtome (Leica, Germany). After permeabilization with 0.3% Triton X-100 (X100PC, Sigma-Aldrich, United States) and blocking with the goat serum (AR0009, BOSTER, China), sections were incubated with primary antibodies for FOXO3 (12829, CST, United States; diluted 1:400), RUNX3 (sc-376591, Santa Cruz, United States; diluted 1:100), CD31 (FAB3628G, R&D Systems, United States; diluted 1:100), CD31 (3528, CST, United States; diluted 1:400), F4/80 (14-4801-82, eBioscience, United States; diluted 1:100), COL1 (72026, CST, United States; diluted 1:100), CD44-PE (553134, BD Pharmingen, United States; diluted 1:100), CD20 (sc-7735, Santa Cruz, United States; diluted 1:100), ANGPTL (sc-365146, Santa Cruz, United States; diluted 1:100), and ITGB-FITC (11-0299-42, eBioscience, United States; diluted 1:100) at 4°C for 14 h. Fluorescence-conjugated secondary antibodies were then stained at 37°C for 1 h, followed by DAPI staining for 10 min or directly sealing slides with AntiFade Mounting Medium (HY-K1042, MedChemExpress, United States). The fluorescence images were captured with CLSM (Nikon, Japan).

## 3 Results

### 3.1 Profiling at the single-cell level depicts a landscape of human dental follicle cell population

**FIGURE 2:**
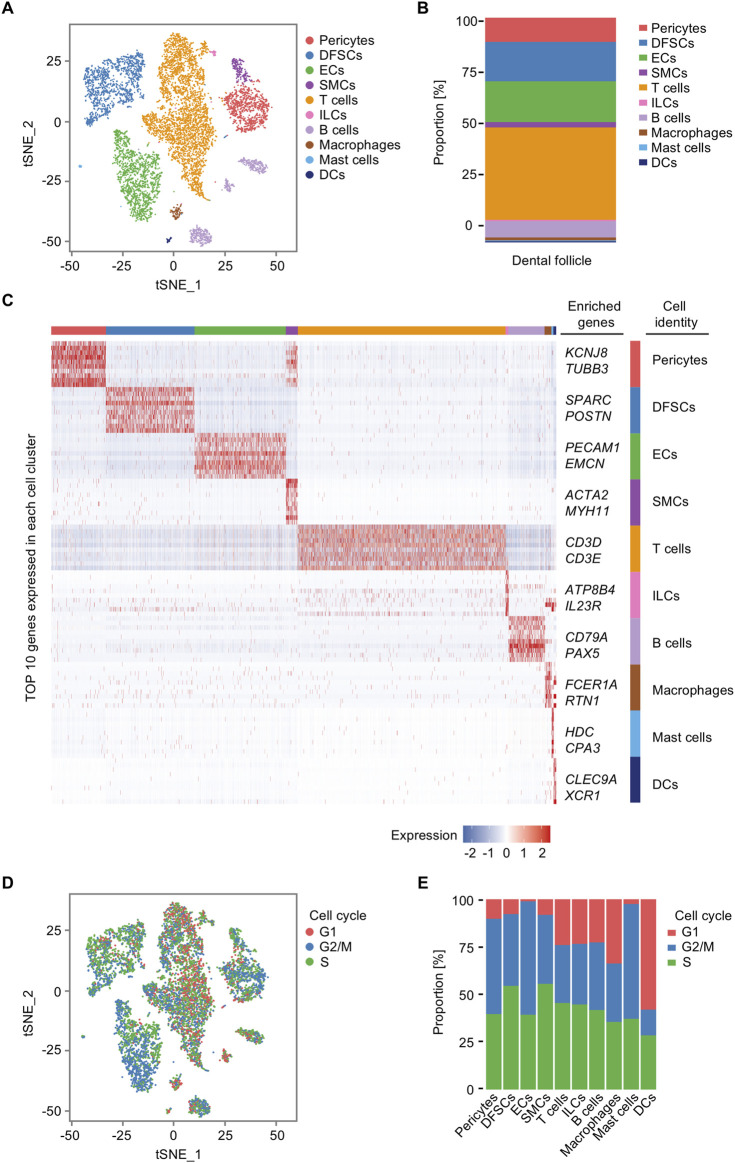
Single-cell transcriptomic profiling of the human dental follicle tissue. **(A)** t-distributed stochastic neighbor embedding (t-SNE) plot of 9,947 cells from the dental follicle colored by 10 cell-type annotations. **(B)** Bar plot of the relative abundance of different cell types in the dental follicle. **(C)** Heatmap of expression of top 10 genes in each cell cluster. **(D)** t-SNE plot of cell cycle analysis for all cells colored by G1, G2/M, and S annotations. **(E)** Bar plot of cell cycle states of each cell cluster in the dental follicle.

At the beginning, we analyzed the diversification of cell populations in the representative postnatal human dental follicle. Through unsupervised marker analysis and manually rectified based on the characteristic expression genes, cells were identified into 10 clusters, including pericytes, dental follicle stem cells (DFSCs), endothelial cells (ECs), smooth muscle cells (SMCs), T cells, innate lymphoid cells (ILCs), B cells, macrophages, mast cells, and dendritic cells (DCs) (Figure 2A). Relative abundance of cell populations revealed an enrichment of T cells and B cells, along with ECs, pericytes, and DFSCs, as the main cell types in the dental follicle tissue (Figure 2B). The large proportion of T cells indicated that although the third molar is in an impacted state, immune cells in the dental follicle tissue may not be in silence and still be activated to assist in the tooth eruption. The profiles of gene expression were displayed as follows: *potassium inwardly rectifying channel subfamily J member 8 (KCNJ8)* and *tubulin beta 3 (TUBB3)* for pericytes (Ando et al., 2022), *secreted protein acidic and cysteine rich (SPARC)* and *periostin (POSTN)* for DFSCs (Shi et al., 2023; Wei et al., 2023), *platelet endothelial cell adhesion molecule 1 (PECAM1)* and *endomucin (EMCN)* for ECs (Cheung et al., 2015; Zhang et al., 2018; Zahr et al., 2016), *actin alpha 2 (ACTA2)* and *myosin heavy chain 11 (MYH11)* for SMCs (Yuan, 2015; von Kleeck et al., 2021), *CD3D* and *CD3E* for T cells (Liu et al., 2024), *ATPase phospholipid transporting 8B4 (ATP8B4)* and *interleukin 23 receptor (IL23R)* for ILCs (Sewell and Kaser, 2022; Croft et al., 2022), *CD79A* and *paired box 5 (PAX5)* for B cells (Küppers and Bräuninger, 2006; Xue et al., 2016), *Fc epsilon receptor Ia (FCER1A)* and *reticulon 1 (RTN1)* for macrophages (Dong et al., 2022), *histidine decarboxylase (HDC)* and *carboxypeptidase A3 (CPA3)* for mast cells (Takai et al., 2019; Atiakshin et al., 2022), and *C-type lectin domain-containing 9A (CLEC9A)* and *X-C motif chemokine receptor 1 (XCR1)* for DCs (Caminschi et al., 2008; Heger et al., 2023) (Figure 2C). Aside from the well-known markers in cell populations, we first defined based on the top 10 genes expressions that novel cell markers like *RTN* for macrophages, *ATP8B4* for ILCs, and *TUBB3* for pericytes. The proliferation activity of each cluster differed slightly (Figure 2D), and the ratio of cells in the S phase in DFSCs and SMCs was higher compared to that of other cell types (Figure 2E). The S phase is the stage of the cell cycle where DNA replication occurs (Bertoli et al., 2013); therefore, a higher proportion of DFSCs and SMCs in the S phase indicated that these cell populations have a greater proliferative potential and play a more important role in tissue regeneration. Taken together, these results identify the cellular composition and cell-specific states in the human dental follicle.

### 3.2 Gene regulatory networks (GRNs) are identified to dictate the biological processes in the adult dental follicle

**FIGURE 3:**
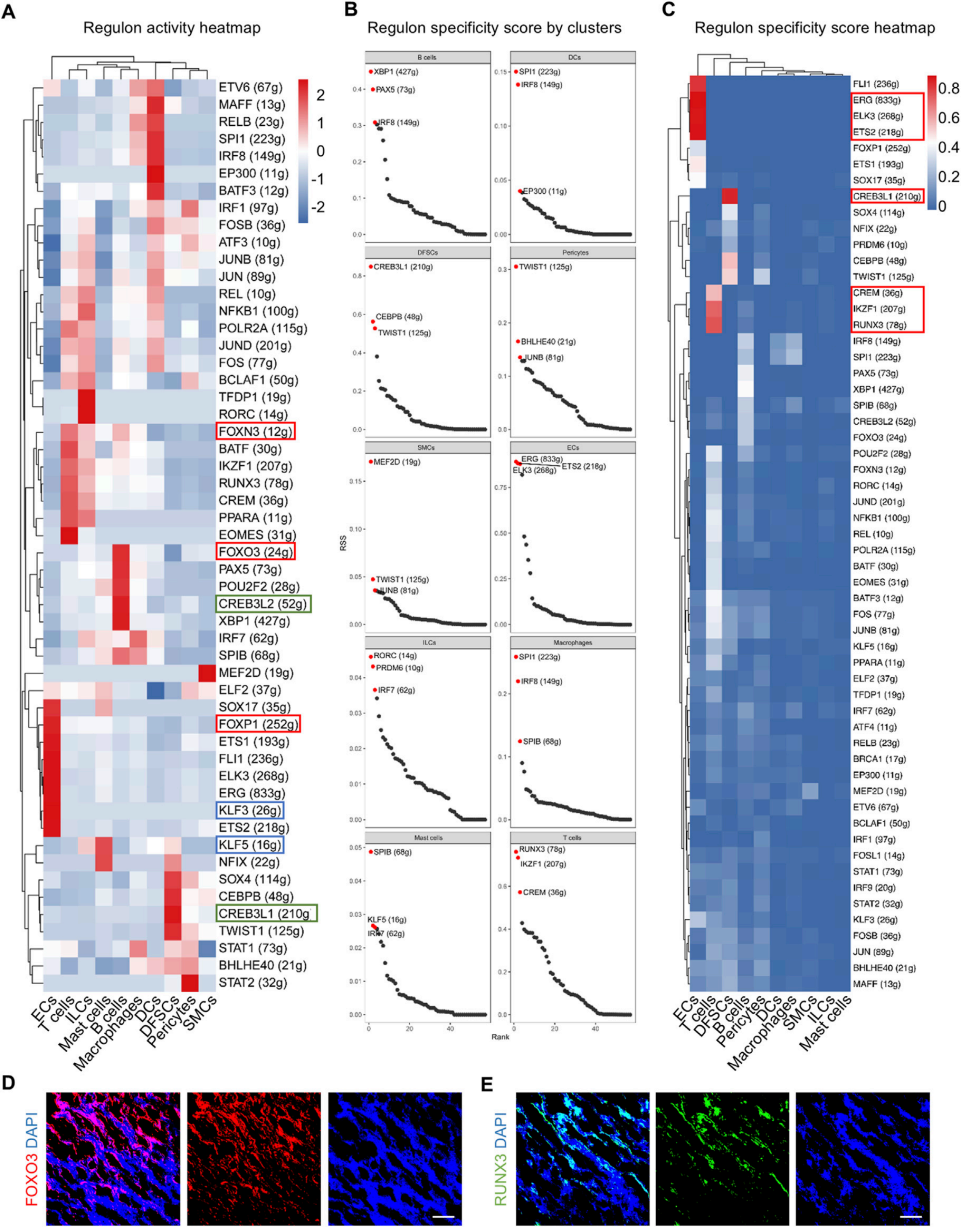
Identification of major regulons in the human dental follicle by SCENIC. **(A)** Heatmap of regulon activity score (RAS) for each cell cluster. **(B)** Scatter plot of regulon specificity score (RSS) for the regulons with the highest specific correlation in each cell cluster. Red dots indicating the top three regulons with the highest RSS. **(C)** Heatmap of RSS in each cell cluster. **(D)** Immunofluorescence images showing validation of FOXO3 expressed in the dental follicle. Scale bar, 100 μm. **(E)** Immunofluorescence images showing validation of RUNX3 expressed in the dental follicle. Scale bar, 100 μm.

GRNs play a critical role in the regulation of cell activity. A collection of genes called regulon is regulated as a unit by the same regulatory element, typically a transcription factor (TF). SCENIC analysis was employed to depict the multiple cell type-specific regulons, thus comprehensively constructing GRNs in the dental follicle. In addition, regulon analysis has two metrics including the regulon-specific score (RSS) and the high regulon activity score (RAS). The RSS measures the specificity of a regulon to a particular cell type, indicating how unique the expression of a regulon is to that cell type. It is crucial for identifying cell type-specific regulatory programs. The RAS measures the activity of a regulon across cells, providing insights into which regulatory programs are active in different cellular contexts. As a result, TFs with high RAS were discovered in cell clusters in the adult dental follicle. For example, Forkhead box (FOX) members, such as FOXN3, FOXO3, and FOXP1, were highly enriched in ECs, T cells, and B cells, indicating that immune and vascular components were determined to regulate postnatal dental follicle development, functioning like the other FOX family members indispensable for the differentiation of periodontal tissue (Jing et al., 2022). Furthermore, CAMP responsive element-binding protein (CREB) family members, including CREB3L2 and CREB3L1, were enriched in DFSCs and B cells, and Kruppel-like factor (KLF) families, such as KLF3 and KLF5, were enriched in ECs and mast cells, also suggesting cross-cell-type regulations between vascular cells and immune cells (Figure 3A). Specific regulons to each cell cluster were also identified based on the RSS (Figure 3B). Especially, regulons of ECs were among the most specific in cell populations, such as E-26 transformation-specific (ETS) transcription factors (Figure 3C). T cells were specifically governed by IKAROS family zinc finger 1 (IKZF1) and Runt-related transcription factor 3 (RUNX3), and DFSCs were governed specifically by CREB3L1 (Figure 3C). It is inferred that CREBL3L might regulate B cells and DFSCs to participate in periodontal bone tissue differentiation under the immune responses. Immunofluorescence images confirmed the FOXO3 and RUNX3 expressed widely in the dental follicle (Figures 3D, E). Collectively, GRNs are identified in the postnatal human dental follicle, which dictate the complex cellular activities.

### 3.3 Regulon modules are classified to synergistically perform specific functions

**FIGURE 4:**
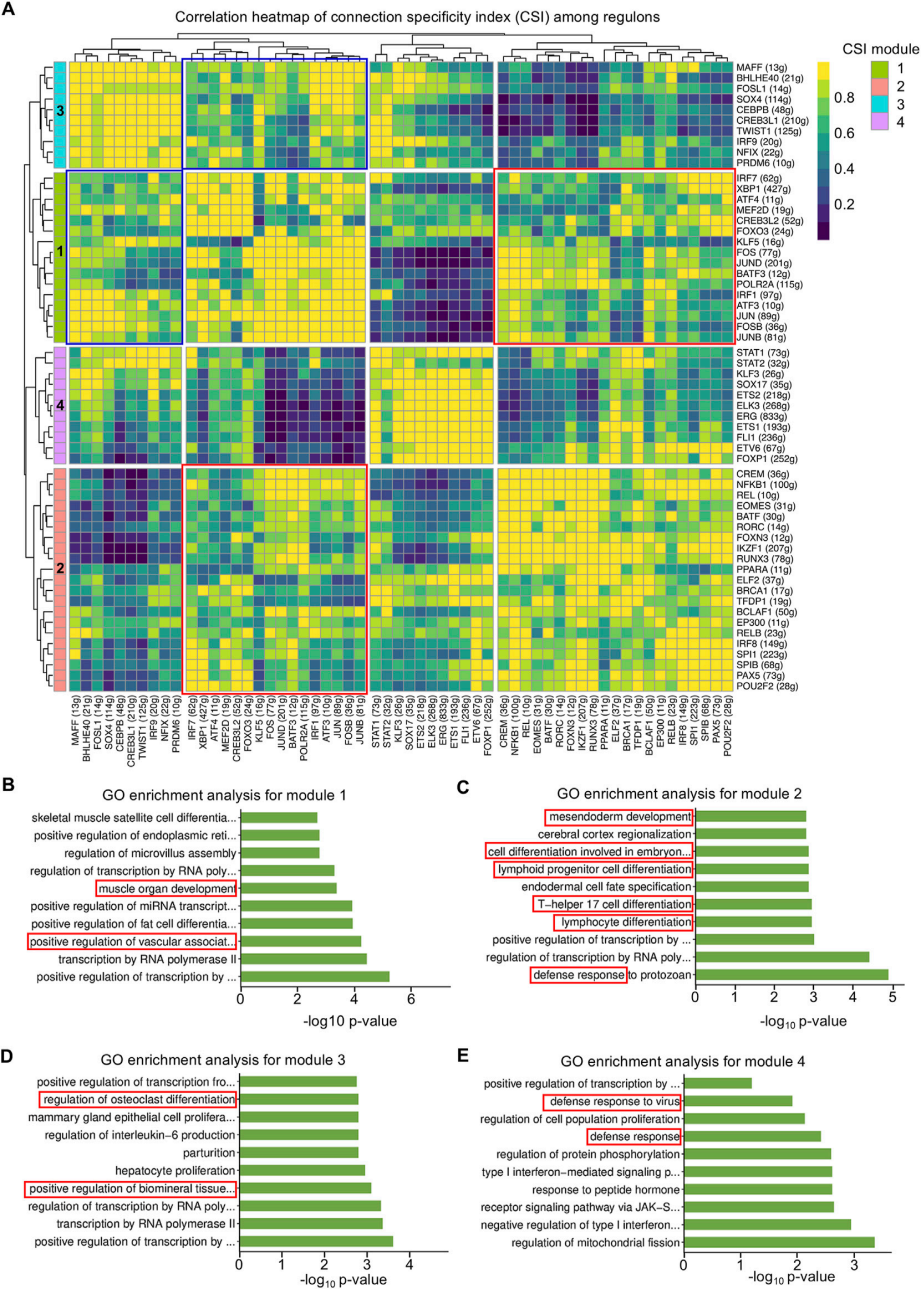
Regulon modules function in similar modes to regulate the downstream effect. **(A)** Matrix of inter-regulon expression correlation analysis showing four modules according to the connection specificity index (CSI). **(B)** Bar plot of Gene Oncology (GO) enrichment analysis for transcription factors (TFs) in module 1. **(C)** Bar plot of GO enrichment analysis for TFs in module 2. **(D)** Bar plot of GO enrichment analysis for TFs in module 3. **(E)** Bar plot of GO enrichment analysis for TFs in module 4.

Next, regulons were divided into four modules according to specific functions. The high connection specificity index (CSI) provides information about the co-regulation and potential collaboration of different regulons. Regulons with high CSI values are likely to be involved in similar cellular functions and may co-regulate downstream genes and cell function (Figure 4A). In detail, module 1 contained CSI regulons in roles characterized by muscle organ development and vascular-associated differentiation (Figures 4A, B). Module 2 was involved in regulating mesendoderm development and lymphoid progenitor differentiation (Figures 4A, C). Module 3 was discovered to take part in the mineralization process, as well as osteoclast differentiation (Figures 4A, D). Finally, module 4 was unraveled to contribute to immune responses (Figures 4A, E). In addition, modules 1 and 2 had a close correlation which might reflect the synergistic effect between vascular and immune cell development (Figure 4A), and modules 1 and 3 potentially cooperated to regulate the coupling of vascular and biomineralized activities (Figure 4A). It was supported that VEGF factors secreted by ECs influence the activity of osteoblasts and osteoclasts, further integrating vascular and biomineralized activities (Song et al., 2023). Importantly, vascular ECs influence immune cell migration and activation, while immune cells affect vascular function and remodeling. This interaction is particularly involved in processes such as inflammation, wound healing, and tissue regeneration (Silberman et al., 2021). Together, regulon modules are defined according to biological functions, part of which has concerted actions in the dental follicle tissue.

### 3.4 Communication pattern analysis reveals the interrelationship between immune and vascular niche components

**FIGURE 5:**
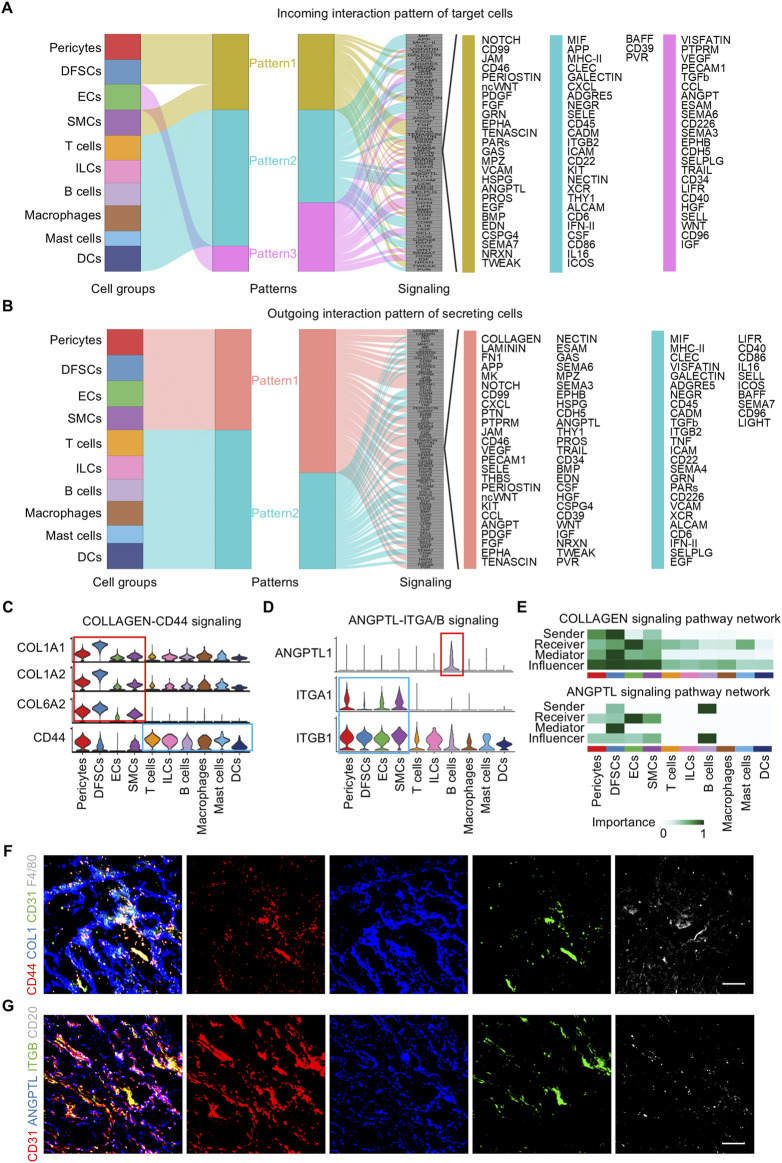
Pattern of cell communication in the human dental follicle. **(A)** Inferred incoming communication pattern of target cells. **(B)** Inferred outgoing communication pattern of secreting cells. **(C)** Expression distribution of COLLAGEN signaling genes. **(D)** Expression distribution of ANGPTL signaling genes. **(E)** Heatmap of the relative importance of each cell cluster based on the computed network centrality measures of COLLAGEN and ANGPTL signaling. **(F)** Immunofluorescence images showing validation of COL1 (blue), CD31 (green), F4/80 (gray), and CD44 (red) in dental follicles. Scale bar, 100 μm. **(G)** Immunofluorescence images showing validation of CD31 (red), ANGPTL (blue), ITGB (green), and CD20 (gray) in dental follicles. Scale bar, 100 μm.

Cell–cell communication patterns refer to the various ways in which cells interact and communicate with each other, which is essential for the coordination of cellular activities in multicellular organisms. Outgoing patterns reveal how sender cells (cells as signal sources) coordinate with each other and with certain signaling pathways to transmit messages to target cells. Incoming patterns show how target cells (cells as signal receivers) coordinate with each other and with certain signaling pathways to react to the signals they receive. To explore how multiple cell clusters and signaling pathways collaborate in the dental follicle, CellChat analysis was employed which uncover three communication patterns for the incoming signaling pathway of target cells and two patterns for the outgoing signaling pathway of secreting cells (Figures 5A, B). Specifically, incoming signaling of pericytes, DFSCs, and SMCs was driven by pattern #1, which included pathways including NOTCH, PERIOSTIN, fibroblast growth factor (FGF), angiopoietin (ANGPT), and BMP, which guided angiogenesis and osteogenesis (Figure 5A) (Ramasamy et al., 2014; Rahman et al., 2015). All immune cells were characterized by pattern #2 of incoming signaling, represented by C-X-C motif chemokine ligand (CXCL), Integrin subunit beta 2 (ITGB2), and interferon-II (IFN-II) pathways (Figure 5A), which mediated immune cell activation and migration (Zhang et al., 2021). ECs solely dictated pattern #3 of incoming signaling, including vascular endothelial growth factor (VEGF), PECAM1, and ANGPT pathways (Figure 5A), which were known for their importance in vessel formation (Eklund and Saharinen, 2013; Luo et al., 2023). For outgoing signaling, pericytes, DFSCs, SMCs, and ECs were categorized into pattern #1, while immune cells were grouped into pattern #2 (Figure 5B).

Considering that immune and vascular cells represent critical niche components of the dental follicle tissue and that they were regulated by several overlapping regulons, immune and vascular interactions were further investigated. The inferred ligand–receptor pairs were shown (Figures 5C, D). In detail, the COLLAGEN vascular signaling also participated in launching downstream immune reactions via the CD44 receptor (Figure 5C). In turn, immune cells sent signals to vascular components. B cells were the representative secreting cells of vessel-regulating ANGPTL1 (Figure 5D). CellChat analysis enables the identification of dominant senders, receivers, mediators, and influencers in cell–cell communication networks by multiple network centrality analysis for each cell population. Network centrality analysis of the inferred communication signaling confirmed that ECs were the most prominent source of COLLAGEN and ANGPTL ligands for immunomodulation (Figure 5E). Notably, ECs were also the dominant mediator of regulating immune responses and the receiver of immunoregulatory information (Figure 5E), suggesting its role as a gatekeeper of cell–cell communication in the adult dental follicle microenvironment. Within the immune cells, B cells were the main signal senders functioning in ANGPTL signaling interactions (Figure 5E). Immunofluorescence validated that the reciprocal co-localization of CD44 on macrophages labeled with F4/80 and COL1 secreted by ECs labeled with CD31, as well as ITGB on ECs labeled with CD3 and ANGPTL secreted by B cells labeled with CD20 (Figures 5F, G), strongly confirming the interactions between the aforementioned receptor–ligand pairs and the interactions between ECs and immune cells. Taken together, these results reveal the interrelationship between immune and vascular components of the dental follicle tissue, which potentially regulates the niche homeostasis and responses.

## 4 Discussion

The heterogeneity of cells, cell–cell interactions and their gene regulatory networks construct a complicated but elaborate information landscape. However, the specific mechanisms of coordination among diverse cell populations in the specific cellular components and the intricate regulatory mechanisms governing the postnatal biological activities and function of the dental follicle remain unclarified. In this study, we performed scRNA-seq analysis to reveal the cell composition within the dental follicle tissue and highlight the importance of GRNs and cell–cell interactions in the dental follicle microenvironment. Through a cohort of bioinformatic experiments, we showed the gene regulatory and signaling network underlying cell lineage diversification and crosstalk. These results serve as an important cornerstone for future studies in the mechanisms regulating dental tissue regeneration through regulating immune and vascular reactions.

GRNs are complex networks consisting of interacting molecules that dictate the biological activities in tissues and organs. Different tissues and organs have their own specific GRNs, and these networks control tissue-specific gene expression patterns. Previous studies have identified that GRNs are pivotal for tooth development and patterning in the enamel knot of the dental epithelium and dental mesenchyme, especially known as Eda signaling, DLX signaling, and FOX signaling (Jing et al., 2022; Tucker et al., 2000). In this study, we have focused on GRNs in the adult dental follicle across the human third molar for the first time. Consistent with previous findings, we revealed that FOX, KLF, and CREB3L families function importantly in the tissue homeostasis through regulating DFSCs, ECs, T cells, and B cells in the dental follicle (Lam et al., 2013). In the view of evolutionary conservativeness of FOX across different species, we complement that FOX families are crucial for regulating a wide variety of biological functions both in mouse dental development and in the human postnatal dental follicle (Golson and Kaestner, 2016). However, it is controversial that FOXO1 is beneficial or detrimental for alveolar bone reconstruction. On one hand, patients suffered from periodontitis had a low expression of FOXO1 and the combination of FOXO1 to METTL3 regulates PDLSCs to promote osteogenesis through the PI3K/AKT signaling pathway (Wang Q. et al., 2023). On the other hand, FOXO1 deficiency greatly rescued osteoblast differentiation and prevented the progression of age-related alveolar bone resorption (Wang et al., 2023b). On the basis of SCENIC analysis, we uncover that FOXN3 and FOXO3 may play vital roles in immune cell regulation in the dental follicle tissue (Zhu et al., 2022), FOXP1 might function to affect the vascular microenvironment. In addition, CREBL3L might participate in periodontal bone tissue differentiation in accordance with its ability to promote bone morphogenesis and regulate the expression of hypoxia-inducible factor-1α (HIF-1α) improving bone angiogenesis (Zhao et al., 2024; Cao, 2021). Furthermore, we found that FOXO3 is expressed widely in the dental follicle, which meant its large requirement in maintaining tissue homeostasis. Nevertheless, the specific effect of FOX families within adult dental follicle should be emphasized deeply so as to achieve precise regulation for medical practice. Accordingly, systematic explanations for GRNs contribute to understand the key mechanisms of coordinated and orderly regulation of biological process, which also enlighten potential new strategies for regenerative medicine and disease treatment (Sadier et al., 2020).

Noticeably, various immune cells are involved and exert important functions, yet their specific signaling of function remains poorly understood. A previous report has analyzed human tooth germs comparatively at two different stages at the single-cell level, which indicates T cells, neutrophils, and macrophages as the main immune cell types in human developing tooth germs (Shi et al., 2021). In line with their findings, we also defined various immune cells within the dental follicle at the postnatal stage, and particularly T cells had the largest proportion. However, intercellular interaction patterns are only focused on the limited subpopulations of T cells or non-T immune cell subpopulations, revealing that T cells exhibited strong communication with osteoclasts specifically by signals from CCL3/CCL4/CCL5 to CCR1/CCR5 (Shi et al., 2021). Complementary to the communication pattern contributing to bone resorption, we provided a novel perspective on the interaction relationship between immune cells and vascular cells, on the basis of collaborated CSI regulon modules 1 and 2 potentially regulating their development. The recruitment of immune cells has been underscored remarkably playing a role in inflammatory responses, angiogenesis, and tissue remodeling (Zarubova et al., 2022). The collaboration of neutrophils and macrophages leads to a sufficient revascularization of implanted biomaterials (Lin et al., 2017). Moreover, M2 macrophages produce MMP-9 to remodel the ECM and release proangiogenic factors like VEGF, TGF-β, and PDGF important for vascular cell recruitment and consequent blood vessel stabilization (Ebrahem et al., 2010; Kwee and Mooney, 2015). Recently, proangiogenic macrophages (PraM), a new subpopulation in macrophages, has been found widely distributed around vessels in multiple organs and play an important role in regulating angiogenesis (Wang et al., 2023c). In addition, monocytes have been recognized as the pioneer to lead and prepare a favorable microenvironment for angiogenesis through migrasomes enriched in angiogenic factors (Zhang et al., 2022). However, there is still lack of evidence about vascular microenvironment modulating the formation or recruitment of immune cells during human tissue repair. In our study, ligand–receptor pair analysis and immunofluorescence staining outcome indicated that ECs are co-localized with macrophages. Collagen as a major component of the ECM of the vessel wall is not only crucial for maintaining vascular integrity and elasticity (He et al., 2022), but it can be inferred that COL1 originating from ECs interacted with receptor ITGB on the macrophages and induced reciprocal recruitment to form an assembly. Importantly, the ECM could shape immune cell phenotypes (Jürgensen et al., 2020), beneficial for regeneration accompanied by modulation of the immune response in the host (Xie et al., 2021). Our analysis also discovered the role of B cells communicating with ECs through ANGPTL-ITGA/B ligand–receptor pairs in regeneration promotion effect. In accordance with previous findings, it has been illuminated that mature naïve B cells enhance angiogenesis and accelerate tissue regeneration by either secreting pro-angiogenic mediators such as VEGF or TGF-β (Yang et al., 2013; Iwata et al., 2009). Future research studies are still necessitated to provide direct evidence to validate the vascular cells regulating the immune cells when applied to tissue regeneration with target regulation.

It should be noted that there is variability in the sample collecting stages. Despite our efforts to select samples that met similar criteria, the fact that they were not acquired completely homogenous since tooth developmental stages exhibit heterogeneity, and it is difficult to ensure all eligible samples collected simultaneously could introduce biases into our analysis. This non-uniformity may have limitations on detecting lowly expressed genes, potentially leading to the loss of significant information. Furthermore, although our study detected a substantial number of immune cells and explored their interactions with vascular cells, the possibility of inflammatory infiltration in the dental follicle cannot be overlooked. Despite these limitations, our study provides valuable insights into the complex interplay between immune cells and vascular components within the adult dental follicle. We hope that future research studies can build upon the conclusions drawn in this study to conduct further validation experiments, thereby obtaining a more comprehensive landscape of vascular and immune components in the dental follicle tissue.

## 5 Conclusion

This study depicts a detailed landscape of GRNs and cell–cell communication networks in the adult human dental follicle. Ten cell clusters are revealed by dimension reduction and clustering annotation. A series of complex GRNs and cell–cell communication networks are uncovered by SCENIC and CellChat analysis. Significantly, we emphasize the close connection between vascular and immune cells regulated by a similar set of regulons involved in vascular and immune cell development, specifically COLLAGAN-CD44 ligand–receptor pairs and ANGPTL1-ITGA/ITGB ligand–receptor pairs constructed the information bridge between immune cells and vascular cells, potentially promoting angiogenesis and immunoregulatory effect during tissue regeneration. These findings will inspire further application of dental follicle tissue in tissue engineering regeneration, as well as the potential targets for regulating the regenerative microenvironment through the mutual modulation of vessels and immunity.

## Data Availability

The datasets presented in this study can be found in online repositories. The names of the repository/repositories and accession number(s) can be found at: https://ngdc.cncb.ac.cn/search/specific?db=hra&q=HRA008022.
